# Tissue-Specific Immunity in Homeostasis and Diseases

**DOI:** 10.1155/2019/7616509

**Published:** 2019-10-15

**Authors:** Ning Wu, Erwei Sun, Dongfang Liu, Di Yu

**Affiliations:** ^1^Department of Immunology, School of Basic Medicine, Tongji Medical College, Huazhong University of Science and Technology (HUST), Wuhan, China; ^2^Department of Rheumatology and Immunology, The Third Affiliated Hospital, Southern Medical University, Guangzhou, China; ^3^Department of Rheumatology and Immunology, Shunde Hospital, Southern Medical University, Foshan, China; ^4^Department of Pathology, Immunology and Laboratory Medicine, Rutgers University-New Jersey Medical School, 185 South Orange Avenue, Newark, NJ 07103, USA; ^5^Center for Immunity and Inflammation, New Jersey Medical School, Rutgers-The State University of New Jersey, Newark, NJ 07101, USA; ^6^Department of Immunology and Infectious Diseases, The John Curtin School of Medical Research, The Australian National University, Acton, ACT, Australia

Substantial evidence supports the critical role of immune responses in nonlymphoid tissues, including the skin, liver, lung, joint, gut, and tumor [1, 2]. Both innate and adaptive immune cells residing in these tissues regulate local homeostasis and contribute to tissue-specific diseases [3, 4]. Recent reports have revealed distinct functions of tissue-specific immunity under physiological and pathological conditions, as well as its great potential as a target in clinical applications [5, 6]. However, the underlying mechanisms remain poorly understood, and more tissue-specific scenarios need to be explored. In this special issue, we gathered a serial of research and review articles focusing on tissue-specific immunity in homeostasis and diseases.

Systemic autoimmune diseases include a wide range of disorders, such as rheumatoid arthritis (RA) and systemic lupus erythematosus (SLE), with the production of autoantibodies as a hallmark [7]. These diseases target nonlymphoid tissues and cause severe tissue damage as major complications. Several articles in this special issue provide new insights into autoimmune diseases. In order to identify biomarkers for RA, C. Rossato et al. describe the correlation between the early production of peritoneal chemokine and the susceptibility to arthritis using a pristane-induced arthritis mouse model, which may serve as potential new biomarkers for RA. M. J. Gutierrez et al. report antibody-secreting cells in the peripheral blood that served as clinical biomarkers to predict infectious risk in patients with RA. Y. Du et al. discover that CD38 is required in collagen-induced arthritis through the NF-*κ*B signaling pathway, suggesting that CD38 represents a potential therapeutic target in the treatment of arthritis. Moreover, by analyzing data of single-cell RNA sequencing (scRNA-seq), S. Cai et al. find that the transition of synovial fibroblasts in rheumatoid arthritis and osteoarthritis shares similar processes. Besides arthritis, L. Duan et al. evaluate the roles of CD14, either membrane-bound or soluble, in patients with gout. R. Witas et al. summarize the functions of TAM receptors in innate immune cells and their role in the development of Sjogren's syndrome.

Apart from autoimmune diseases, several other exciting aspects of tissue-specific immunity have been included in this special issue, especially the interaction between microbiota and immune cells, an emerging critical factor in human diseases [8]. O. V. Kovaleva and colleagues discuss how the microbiome interrupts the immune environment in the lung, leading to malignant tumor progression in patients. Immunity in the female reproductive tract, the cervix, is reviewed by J. B. De Tomasi and colleagues. An unbalanced cervical immune niche contributes to female reproductive disorders. Moreover, R. Slutsky et al. report the existence of decidual exhausted and senescent T cells, which is related to term labor. F. Ohori et al. describe that orthodontic tooth movement-induced TNF-*α* production could trigger the expression of sclerostin and result in enhanced osteoclast formation on the compression side in the mouse model. Neuroimmune crosstalk is another emerging area implicated in development and diseases [9]. The contribution of the monocyte-derived macrophage to diabetic neuropathy discovered by J.-J. Sun et al. is collected in this special issue. Using machine learning, N. D. Macedo et al. develop a method to detect VEGF in renal tissue sections automatically.

In addition to basic research articles, clinical evidence signifies tissue-specific immunity in human diseases. A clinical study by Y. Tang et al. discovered that the *rs744166* allele of *STAT3* is associated with increased STAT3 tyrosine phosphorylation and higher IL-23 secretion by innate lymphoid cells (ILCs). As a result, Crohn's disease patients bearing this risky allele demonstrated prolonged inflammation. Autoantibodies usually cause tissue damage in autoimmune diseases. Intriguingly, R. Mills et al. find that autoantibodies reactive to HSP72 correlated to improved idiopathic pulmonary fibrosis in patients. However, further study is required to clarify the underlying mechanism. Q. Song et al. analyze Chinese patients with invasive ductal breast cancer and find that the number of CD8/PD-1 tumor-infiltrating lymphocytes is associated with their clinical and pathologic characteristics.

Tissue-specific immunity includes vast fields in clinical and basic research. The current issue only covers a slice of topics, but it already reports many new discoveries and important hypotheses. We believe this special issue provides us new clues and will continuously motivate the community to investigate this important topic in the future.
